# Antioxidant Activity of β-Carotene Compounds in Different *in Vitro* Assays 

**DOI:** 10.3390/molecules16021055

**Published:** 2011-01-25

**Authors:** Lars Muller, Volker Bohm

**Affiliations:** Institute of Nutrition, Friedrich Schiller University Jena, Dornburger Straße 25-29, 07743 Jena, Germany

**Keywords:** β-carotene isomers, β-apo-carotenoids, ferric reducing, ABTS bleaching, peroxyl radical scavenging

## Abstract

β-Carotene (BC) is the most abundant carotenoid in human diet, almost solely as (*all-E*)-isomer. Significant amounts of (*Z*)-isomers of BC are present in processed food as well as in mammalian tissues. Differences are described for the activity of various BC isomers in forming retinal and protecting against cancer and cardiovascular diseases. Eccentric cleavage of BC leads to degradation products such as carotenals. A variety of negative consequences were published for the non-vitamin A active BC metabolites, such as inducing the carcinogenesis of benzo[a]pyrene, impairing mitochondrial function, or increasing CYP activity. To increase the knowledge on the antioxidant activity, a variety of BC isomers and metabolites were tested in various *in vitro* assays.

In the present study, no ferric reducing activity (FRAP assay) was observed for the BC isomers. Between the major BC isomers (*all-E*, *9Z*, and *13Z*) no significant differences in bleaching the ABTS^●+^ (αTEAC assay) or in scavenging peroxyl radicals (ROO^●^) generated by thermal degradation of AAPH (using a chemiluminescence assay) were detected. However, the (*15Z*)-isomer was less active, maybe due to its low stability. The degradation to β-apo-carotenoids increased FRAP activity and ROO^●^ scavenging activity compared to the parent molecule. Dependence on chain length and character of the terminal function was determined in αTEAC assay with following order of increasing activity: β-apo-8’-carotenal < β-apo-8’-carotenoic acid ethyl ester < 6’-methyl-β-apo-6’-carotene-6’-one (citranaxanthin). The results indicate that BC does not lose its antioxidant activity by degradation to long chain breakdown products.

## Introduction

**Figure 1:**
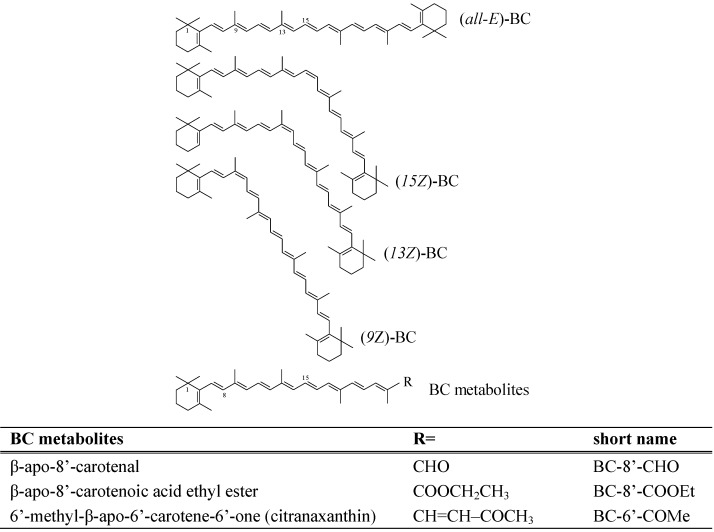
Structures of analyzed β-carotene (BC) isomers and metabolites.

Carotenoids are a widespread group of naturally occurring fat-soluble colorants. In developed countries, 80-90% of the carotenoid intake comes from fruit and vegetable consumption. Of the more than 700 naturally occurring carotenoids identified thus far, approx. 50 are present in the human diet and can be absorbed and metabolized by the human body [1]. However, only six of them (β-carotene, β-cryptoxanthin, α-carotene, lycopene, lutein and zeaxanthin) account for more than 95% of total blood carotenoids. β-Carotene (BC) is a naturally occurring orange-colored carbon-hydrogen carotenoid, abundant in yellow-orange fruits and vegetables and in dark green, leafy vegetables [2]. It is also the most widely distributed carotenoid in foods [3]. BC undergoes *trans* (*E*) to *cis* (*Z*) isomerization [4], whereas the (*all-E*)-form is the predominant isomer found in unprocessed carotene-rich plant foods [5,6]. Food processing or long-term storage of carotenoid-rich vegetables can lead to degradation and/or isomerization of carotenoids [1,7]. Although low concentrations are found in circulating human serum, BC (*Z*)-isomers are present in human tissues where it is expected to exert their biological function(s) [8]. Significant amounts of (*9Z*)-, (*13Z*)-, and (*15Z*)-isomers of BC were found in liver, kidney, adrenal gland and testes up to 25% of the total BC, whereas in human serum (*all-E*)-BC was the dominant isomer with 95% of the total BC amount [9]. Chemical structures of the main BC isomers found in food and human tissues are shown in Figure 1.

Nutrition has a significant role in the prevention of many chronic diseases such as cardiovascular diseases (CVD), cancers, and degenerative brain diseases [10]. The consumption of food-based antioxidants like BC seems to be useful for the prevention of macular degeneration and cataracts [11]. Numerous epidemiological studies have suggested an inverse relationship between intake of BC, fruits and vegetables, particularly raw fruits and vegetables and dark green, leafy and cruciferous vegetables, and the risk of oesophageal adenocarcinoma and Barrett's oesophagus [12]. Additionally, several studies have observed a protective effect of BC from foods, along with a diet rich in fruits and vegetables, on liver carcinogenesis and lung disease [13,14]. BC has potential antioxidant biological properties due to its chemical structure (see Figure 1) and interaction with biological membranes [15]. It is well-known, that BC quenches singlet oxygen with a multiple higher efficiency than α-tocopherol. [16]. In addition, it was shown that (*Z*)-isomers of BC possess antioxidant activity *in vitro* [17,18,19].

In contrast, three large BC intervention trials: the β-Carotene and Retinol Efficacy Trial (CARET), the Alpha-Tocopherol Beta-Carotene Cancer Prevention Study (ATBC), and the Physician's Health Study (PHS) have all pointed to a lack of effect of synthetic BC in decreasing cardiovascular disease or cancer risk in well-nourished populations up to increased lung cancer incidence and mortality in smokers [14,20,21]. 

In vertebrates, BC is converted into two molecules of retinal, in a reaction catalyzed by β,β-carotene-15,15’-monooxygenase (BCMO I), like other provitamin A carotenoids too [22]. Of the 50 different carotenoids that can be metabolized into vitamin A, BC has the highest provitamin A activity [2]. The formed retinal is further metabolized to the vitamin A derivatives retinoic acid (RA) and retinol. The provitamin A activity of (*Z*)-isomers is much lower than that of (*all-E*)-BC. (*9Z*)-BC has a relative bioconversion to retinol of 38%, (*13Z*)-BC 53% whereas the (*all-E*)-form is 100% [23]. Besides being essential for vision, RA is a major signal pathway controlling molecule which regulates a wide range of biological processes. RA is the ligand of two classes of nuclear receptors, the retinoic acid receptors (RARs) and the retinoid X receptors (RXRs). (*all-E*)-BC is a precursor of (*all-E*)-RA, which preferentially binds to RARs, whereas (*9Z*)-BC is a precursor of (*9Z*)-RA – the preferred ligand for RXRs [24]. 

In addition to this central cleavage pathway, an eccentric cleavage was proposed in healthy mammals after incubation of BC with liver, kidney and lung homogenate of rats, ferrets, and monkeys [25]. By stepwise oxidation from one end of the polyene chain a sequence of β-apo-carotenal derivatives were presumably formed, e.g. β-apo-8’-carotenal (shown in Figure 1). The formed aldehydes were further cleaved to short-chain carbonyl compounds, or converted to β-apo-carotenol, β-apo-carotenoic acids or their esters, or oxidized to retinoic acid by β-oxidation pathways [26,27]. The three apo-carotenoids studied herein are used as colorants in animal feed and human food. β-apo-8’-Carotenal and β-apo-8’-carotenoic acid ethyl ester are present in some fruits and vegetables, though in low amounts [28], and were recently detected in human plasma [27]. 

In addition to the enzymatic cleavage of BC in mammalian metabolism, free radical attack on BC results in the formation of high amounts of cleavage products. For instance, β-apo-8’-carotenal and 6’-methyl-β-apo-6’-carotene-6’-one (citranaxanthin), shown in Figure 1, were identified in minor amounts in intestinal extracts of vitamin A deficient rats [29]. The results of Allija *et al*. [30] indicate a genotoxic potential of BC cleavage products at physiologically relevant levels of BC and its breakdown products. In contrast, BC itself did not induce cytotoxic or genotoxic effects. Furthermore, when BC was supplemented to primary hepatocytes a dose-dependent increase of cleavage products was observed accompanied by increasing genotoxicity [30]. The authors speculated that these results provide strong evidence that BC breakdown products are responsible for the occurrence of carcinogenic effects found in the Alpha-Tocopherol Beta-Carotene Cancer prevention (ATBC) study and the β-Carotene and Retinol Efficacy (CARET) Trial.

In contrast to the physiologically relevant properties, such as influencing cellular signal pathways, gene expression or induction of detoxifying enzymes, the knowledge on antioxidant potential of BC compounds is scarce. Therefore, the aim of the study was to investigate both BC isomers and some non-retinoic metabolites on their antioxidant activity in various *in vitro* assays, compared to another nutritionally relevant substance – vitamin E (α-tocopherol).

## Results and Discussion

It has been known for many years that carotenoids undergo ‘‘bleaching’’ i. e., lose their color, when exposed to radicals or to oxidizing species. This process involves interruption of the conjugated double bond system either by cleavage or by addition to one of the double bonds. Cleavage can be detected by characterizing the products that are formed, which are frequently carbonyls or epoxides [2]. In the present study, four isomers and three metabolites of β-carotene (BC) were analyzed on their antioxidant activity in three different *in vitro* assays. There are at least three possible mechanisms for the reaction of carotenoids with radical species. They include (1) radical addition; (2) electron transfer to the radical; or (3) allylic hydrogen abstraction [2]. 

The ability of BC and its degradation products to undergo single electron transfer-based reactions (SET) was utilized in the analysis of ferric reducing (FRAP) and ABTS^●+^ bleaching (αTEAC) activity. Electron transfer reactions have been reported, resulting in the formation of a carotenoid cation radical (CAR^●+^) [31]. Such a cationic radical of BC or its metabolites is entirely conceivable in the reactions with the ferric ion or the synthetic ABTS^●+^. 

**Figure 2:**
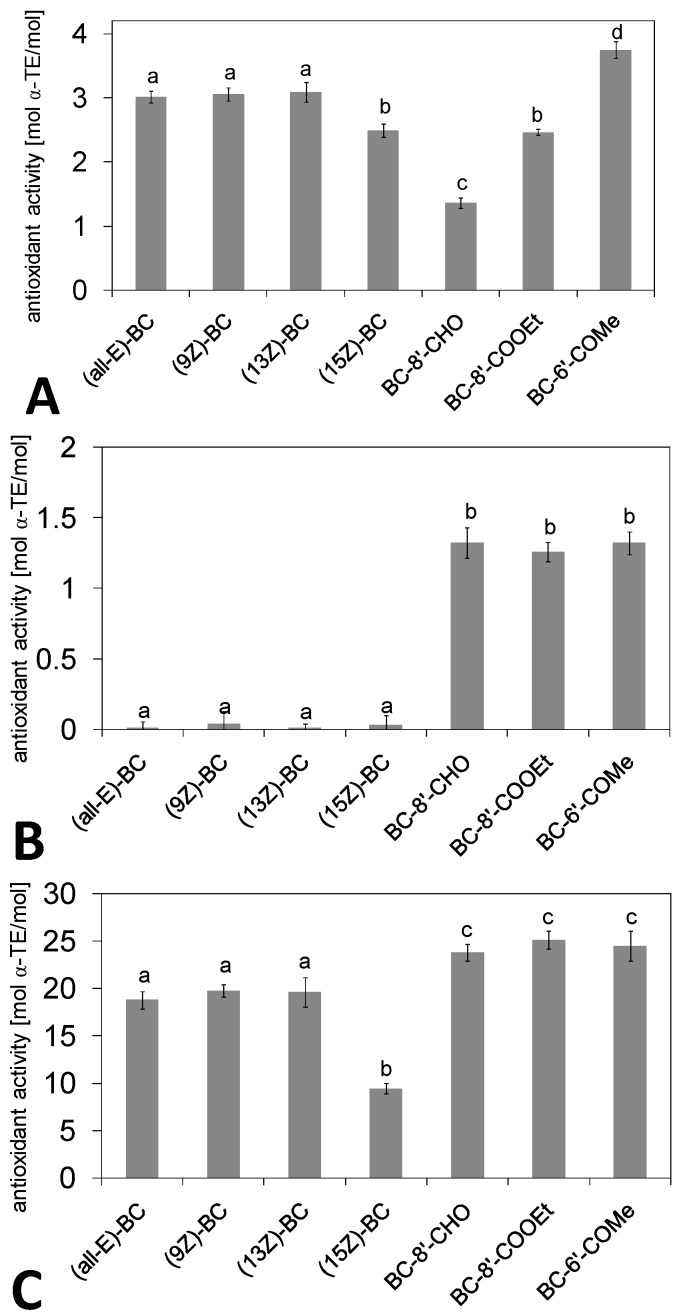
Antioxidant activities (mean ± SD) of β-carotene (BC) isomers and metabolites (at 10 µM) determined by αTEAC (**A**), FRAP (**B**) and CL (**C**) assay with respect to α-tocopherol (α-TE, α-tocopherol equivalents); different superscript letters denote significant differences (ANOVA, *post-hoc* Student-Newman-Keuls, *p* < 0.05). For abbreviations see Figure 1.

In the αTEAC assay, the investigated (*all-E*)-BC and its (*Z*)-isomers showed 3-times higher ABTS^●+^ bleaching activity than α-tocopherol [Figure 2(A)]. The results of Böhm *et al*. showed an antioxidant activity of the BC isomers dissolved in *n*-hexane, marginal higher than that of the calibration compound Trolox^®^. This hydrophilic analogue of α-tocopherol was dissolved in PBS [19]. In the present study, the reference compound α-tocopherol was dissolved in *n*-hexane to be more comparable to the reaction conditions used for the carotenoids. The differences in the reference compound used and in the reaction conditions might have caused the different TEAC values of BC. To date, published results on antioxidant activity of BC isomers *in vitro* differ due to the use of different test systems. Often (*9Z*)-BC was more effective than its (*all-E*)-isomer [17,18,32]. In contrast, there are also investigations under identical conditions which support our results. The studies of Böhm and colleagues showed that the ABTS^●+^ bleaching activity of BC isomers is independent from position of the *cis*-double bond [19]. No significant dependence (*p* > 0.05) of the position of the *cis*-double bond was observed between (*all-E*)-, (*9Z*)-, and (*13Z*)-BC (approx. 3 mol α-TE/mol) in our investigations. However, (*15Z*)-BC displayed a 20% lower activity (2.5 mol α-TE/mol) in this assay (*p* < 0.05). The advanced hindrance between the steric demanding bicyclic carotenoid molecule with a centered *cis*-double bond and the similarly demanding oxidizing agent ABTS^●+^ might have caused this lower activity. The relation of steric demand of ABTS and carotenoids was demonstrated several times [33,34]. Ascorbic acid and phenolic antioxidants like flavonoids, phenolic acids (hydroxylated benzoic acids and cinnamic acids) and tocopherols excite their antioxidant potential by hydroxyl groups at the outer part of the molecule reacting with radicals combined with a conjugated double bond system in vicinity [33,34]. Consequently, a resonance-stabilized radical is formed [37,38]. However, the reactive part of carotenes, like lycopene, α- and β-carotene, is the conjugated polyene chain in the center of the molecule [39]. This fact makes it difficult for steric demanding oxidants to interact with the carotenoid, especially with the bicyclic structures of β-carotene. The results obtained in the FRAP assay, described below, support this hypothesis. Additionally, (*15Z*)-BC is the BC isomer with the lowest stability [40,41] investigated in the present work, due to the higher potential energy of its *cis*-bond. This may have led to a degradation of this isomer during analysis, and consequently a lower antioxidant activity was determined.

Transition metals, such as iron (III) and copper (II), play an important role in the oxidation of LDL *in vitro* as well as *in vivo*, leading to atherosclerosis [42]. BC and other carotenoids have potential antioxidant properties [15], and they were found to be incorporated into LDL particles. However, in our *in vitro* studies, none of the BC isomers showed ferric reducing activity [Figure 2(B)] under the used conditions, which support the findings of Pulido and co-workers [43]. This may be due to the circumstance that the ferric ion is incorporated into the steric demanding *di*-tripyridyltriazine (TPTZ) complex, which was first applied by Benzie and Strain [44]. Our recently published findings using α-carotene, β-carotene, lycopene and a variety of xanthophylls in the FRAP assay supports this hypothesis. It was shown, that lycopene with its acyclic polyene structure showed FRAP activity. The insertion of a hydroxyl function into bicyclic carotenes (leading to e.g. β-cryptoxanthin and zeaxanthin) induced the activity to reduce ferric ions using the TPTZ complex method [34]. The buckle in *cis*-isomers of BC, which may open the molecule to be more assailable to react with large steric demanding oxidants, such as ferric *di*-TPTZ in the FRAP assay, in our case, did not influence the activity of BC.

The noxious effects of an uncontrolled production of oxygen- and nitrogen-centered radicals (ROS, RNS) are amplified by chain reactions (autoxidations), sustained mainly by peroxyl radicals (ROO^●^), that oxidize and alter essential biomolecules such as lipids, lipoproteins, proteins and nucleic acid [45,46]. Krinsky and Johnson [2] proposed that ROO^●^ might add to any place across the polyene chain of a carotenoid, resulting in the formation of a resonance-stabilized, carbon-centered radical (ROO-CAR^●^). Unfortunately, radical-carotenoid reaction products or stabilized carotenoid radicals were not detected *in vivo* to date. Additionally, ROO^●^ can abstract an allylic hydrogen atom at the periphery of the carotenoid, in the case of BC at the 4- and 4’-position [47]. In the present study, the ROO^●^ were formed by thermal degradation of AAPH at 37 °C. The analyzed (*all-E*)-form of BC presented a ROO^● ^scavenging activity, being approx. 20-times higher than that of α-tocopherol [Figure 2(C)]. Hence, β-carotene and its isomers could play a role in the endogenous antioxidant defense system despite their lower concentrations found in human tissues compared to tocopherols. The high scavenging rate found in the present studies supports our recent observations and that of other research groups using typical synthetic ROO^●^ generating azo-initiators such as AAPH, AMVN or AIBN [34,48,49,50,51]. The insertion of a *cis*-double bond at C9 or C13 did not change the antioxidant activity of BC (*p* > 0.05), whereas (*15Z*)-BC (9.5 mol α-TE/mol) was half as active (*p* < 0.05) as (*all-E*)-BC (18.8 mol α-TE/mol) probably caused by oxidative degradation during the analysis as explained for the αTEAC assay above. 

Within the investigated BC metabolites, 6’-methyl-β-apo-6’-carotene-6’-one showed the highest ABTS^●+^ bleaching activity [Figure 2(A)], approx. 4-times higher than α-tocopherol and significantly (*p* < 0.05) higher than its parent molecule (*all-E*)-BC (3.0 mol α-TE/mol), which has the same number of conjugated double bonds (CDB) in the polyene chain. The degradation of BC to β-apo-8’-carotenal and its related carotenoic acid ester led to a significant (*p* < 0.05) decrease of CDB and therefore to a decrease of αTEAC activity. The β-apo-8’-carotenal (1.4 mol α-TE/mol) was only 40% more active than α-tocopherol (1 mol α-TE/mol) and only half as active as (*all-E*)-BC (3.0 mol α-TE/mol), due to its shorter polyene chain system and the electron-withdrawing effect of the carbonyl function. This circumstance causes a higher ionization potential of β-apo-8’-carotenal (4.676 eV) compared to BC (4.414 eV) calculated by Galano [52]. Ionization potentials of compounds are important in SET-based assays such as αTEAC and FRAP assay. In contrast, the change in the terminal function to a carboxylic acid ester with equal chain length led to an comparable activity to (*15Z*)-BC (2.5 mol α-TE/mol), possibly caused by an inductive effect inserted by esterification of the carbonyl function. 

Surprisingly, the breakdown of BC to its metabolites caused a significant increase (*p* < 0.05) of the ferric reducing activity, which supports the hypothesis, that the existence of two non-substituted β-ionone rings has caused the absent FRAP activity of BC. The cleavage of BC to its metabolites, forming a structure with only one β-ionone ring and an oxygenated functional group at the opposite side, led to a significant increase of the ferric reducing activity. The three BC metabolites showed FRAP values being 25% higher than that of α-tocopherol, however, without any significant differences (*p* > 0.05) concerning the length of the conjugated chain or terminal function [Figure 2(B)] of the compounds. 

The three investigated BC breakdown products were highly efficient in preventing luminol oxidation (23.8-25.1 mol α-TE/mol). The degradation of BC to these metabolites led to a significant (*p* < 0.05) increase in the ROO^●^ scavenging activity of approx. 25% [Figure 2(C)]. A significant dependence on chain length or carbonyl related function was not observed (*p* > 0.05). ROO^●^ can abstract a hydrogen atom at each position of the polyene chain [2]. In consequence, the type of terminal function has only significant influence on the reaction between carotenoid and radical if the conjugated double bond system expands. The increase of the activity to scavenge ROO^●^ radicals by insertion of carbonyl functions into the polyene molecule was described several times for BC and its related ketocarotenoids echinenone, canthaxanthin, and astaxanthin [34,49,53]. Carotenoids such as BC can prevent the propagation phase of lipid peroxidation. As known from fatty acid oxidation, the final result of this reaction is degradation of the whole molecule into small polar products. BC should be regarded as peroxidation substrate as well as antioxidative compound [17]. Our studies show that the long-chained non-enzymatic metabolites such as β-apo-8’-carotenal are able to act as substrate in peroxidation and could protect fatty acids from oxidation, too. However, due to the very low amounts of BC metabolites found *in vivo* compared to tocopherols and carotenoids, BC metabolites might be not of relevance for the antioxidant defense system in human organism.

As stressed by Huang *et al.* [54], no single method is adequate for evaluating the antioxidant activity of single compounds or antioxidant capacity of foods or biological samples. Methods based on different mechanistic principles can yield widely diverging results. A variety of methods must be used. In the present study, two different principles were used: αTEAC and FRAP assay measures reducing activity, whereas CL determined ROO^●^ scavenging activity. Standardization is needed by a calculation of the results achieved in the three assays. A simple mathematical treatment is not indicated, because the CL assay gave much higher values due to the low activity of α-tocopherol in this assay. To give no substance in any assay undue preponderance, calculating a global antioxidant activity as a weighted average of the results is necessary [55]. First, the antioxidant activity of the compound detected in the specific method was divided by the average activity of the whole set of compounds by the same method. Afterwards, the calculated values of the specific compounds in each assay were summed and divided by the number of assays used (three in our case). The resulting weighted averages of each compound are given in Table 1 (last column). 

**Table 1:** Antioxidant activities (mol α-TE/mol) of β-carotene isomers and metabolites standardized with respect to α-tocopherol measurement.

	Compound	αTEAC	FRAP	CL	Weighted average
	α-tocopherol	1.0	1.0	1.0	0.7
β-carotene isomers	(*all-E*)-β-carotene	3.0	0.0	18.8	0.8
	(*9Z*)-β-carotene	3.1	0.0	19.8	0.8
	(*13Z*)-β-carotene	3.1	0.0	19.6	0.8
	(*15Z*)-β-carotene	2.5	0.0	9.5	0.5
β-carotene metabolites	β-apo-8’-carotenal	1.4	1.3	23.8	1.3
	β-apo-8’-carotenoic acid ethyl ester	2.5	1.3	25.1	1.5
	6’-methyl-β-apo-6’-carotene-6’-one	3.7	1.3	24.5	1.7
Average		2.5	0.6	17.8	

The right-hand column shows the weighted averages (mol α-TE/mol) obtained by (1) dividing the antioxidant activity of each compound, as determined by the specified method, by the average activity determined for the whole set of compounds by the same method (last row), (2) summing the results of the three assays for the specific compound (αTEAC, FRAP, and CL), and (3) dividing the sum by three.

On this basis, the four analyzed BC isomers showed antioxidant activities comparable to that of α-tocopherol (0.5-0.8 mol α-TE/mol) due to the absent ferric reducing activity of BC-isomers, whereas α-tocopherol displayed a poor CL value. Almost two-times higher activities were observed for the BC breakdown products, with 6’-methyl-β-apo-6’-carotene-6’-one as the most active one (weighted average of 1.7 α-TE/mol).

In addition to our findings on antioxidant activities of BC metabolites, the pro-oxidative effects have to be kept in mind as well. β-apo-8’-Carotenal was shown to be a strong inducer of cytochromes P4501A1 and 1A2 in rat liver, whereas BC itself was not active [56]. Induced cytochrome P450 enzymes could enhance the activation of carcinogens. Oxidative degradation products of BC could also increase the binding rate of benzo[a]pyrene to DNA [57] and may impair mitochondrial function [58,59,60]. And β-apo-8’-carotenal was shown to bound to 2’-deoxyguanosine *in vitro* [61]. In contrast, various beneficial activities were demonstrated *in vitro* for oxidation products of non-provitamin A carotenoids e.g. lycopene [62]. 

## Conclusions

According to our knowledge this is the first study presenting antioxidant activity data of β-carotene (BC) isomers and their metabolites using different types of *in vitro* assays. For the first time, BC related compounds were compared based on their ABTS^●+^ bleaching and ferric reducing activity, as well as on their ROO^●^ radical scavenging activity. All results were compared to the activity of α-tocopherol, which is known as the most active chain breaking and major fat-soluble antioxidant in human tissues. The activity of carotenoids to reduce ferric ions is an important property, because transition metals play an important role in catalyzing LDL oxidation *in vitro* and *in vivo*, leading to atherosclerosis. However, in the present study, ferric reducing activity was detected for BC metabolites, but not for the different BC isomers. Additionally, scavenging activities of the investigated compounds against ROO^●^ generated by thermal degradation of AAPH were 10-25-times higher than that of α-tocopherol. ROO^●^ are important for the initiation of lipid peroxidation chain reactions in food as well as in biological samples. All analyzed BC isomers showed 2.5-3-times higher activity in bleaching ABTS^●+^ than α-tocopherol. Dependence on the antioxidant activity from chain length and terminal group of the β-apo-carotenoids was only observed in the activity of bleaching ABTS^●+^, but not in the more *in vivo* relevant activities like reducing ferric and scavenging ROO^●^. The results of the different assays were summarized by calculating a weighted average for each BC compound to get an overall impression of the antioxidant potential. On this basis, the global antioxidant activity of the BC isomers was comparable to that of α-tocopherol. The activity of breakdown products of BC was twice as high.

## Experimental

### General

2,2´-Azinobis(3-ethylbenzothiazoline-6-sulphonic acid) diammonium salt (ABTS), K_2_S_2_O_8_, and 2,4,6-tripyridyltriazine (TPTZ) were obtained from Sigma-Aldrich (Taufkirchen, Germany). 2,2´-Azobis(2-amidinopropane) dihydrochloride (AAPH) was obtained from Acros Organics (Schwerte, Germany). Luminol was purchased from Fluka (Buchs, Switzerland). DL-α-Tocopherol was purchased from Calbiochem (Darmstadt, Germany) with a purity of 100% shown by GC. β-Carotene (BC) isomers, 6’-methyl-β-apo-6’-carotene-6’-one (citranaxanthin), β-apo-8’-carotenal and β-apo-8’-carotenoic acid ethyl ester were obtained from CaroteNature (Lupsingen, Switzerland) with a purity of 97-99% by HPLC. All solvents used, such as *tert*-butyl methyl ether (TBME) or dimethyl sulfoxide (DMSO), were of HPLC grade. HPLC grade water (18 MΩ) was prepared using a Millipore Milli-Q purification system (Millipore GmbH, Schwalbach, Germany). Buffer salts for phosphate buffered saline (PBS), borax buffer and acetic acid buffer and all other chemicals were of analytical grade.

### Equipment

An ABTS^●+^ solution was prepared in phosphate buffered saline (PBS, 75 mM, pH 7.4) to measure the activity of the BC compounds to bleach ABTS^●+^ in the αTEAC (α-tocopherol equivalent antioxidant activity) assay as described in several publications [19,33,63]. To determine the ferric reducing antioxidant power (FRAP) of BC and its derivatives, a FRAP reagent was prepared as recently described [63,64]. The analysis of the ROO^●^ radical scavenging activity in a chemiluminescence (CL) based assay followed the descriptions as published recently [61]. A luminol solution in DMSO+borax buffer (80+20, v/v) as well as an AAPH solution in DMSO+PBS (80+20, v/v) was prepared daily fresh, and cooled until analysis. Stock solutions of (*all-E*)-BC, its (*Z*)-isomers and metabolites were prepared by dissolving the compounds in toluene+cylohexane (1+4, v/v) to concentrations of 150 µmol/L. A 2.5 mmol/L α-tocopherol stock solution was prepared in ethanol. All stock solutions were stored at -25 ± 2 °C. Prior to analysis, aliquots of the stock solutions were transferred into reaction tubes and the solvent was removed under nitrogen at 30±1 °C in darkness. The residues were immediately dissolved in *n*-hexane (for the use in FRAP and αTEAC assay) or *tert*-butyl methyl ether (TBME)+DMSO (1+9, v/v) for the application in the CL assay. Concentrations of the compounds were adjusted to 100 µmol/L by spectrophotometrical determination using the absorptivity values (*E*_1 %, 1 cm_) at the specific wavelengths listed in Table 2.

**Table 2:** Absorptivity values at specific wavelength maxima in specific solvent, and solvent used for stock solutions of analyzed β-carotene isomers and metabolites and α-tocopherol [65,66,67,68].

Compound	Solvent	Wavelength (nm)	Absorptivity value ( *E*_1%,1 cm_)		Solvent used for stock solution
( *all-E*)-β-carotene	*n*-hexane	453	2592	T/CH (1+4, v/v)	
( *9Z*)-β-carotene	*n*-hexane	445	2550	T/CH (1+4, v/v)	
( *13Z*)-β-carotene	*n*-hexane	443	2090	T/CH (1+4, v/v)	
( *15Z*)-β-carotene	*n*-hexane	447	1820	T/CH (1+4, v/v)	
β-apo-8’-carotenal	ethanol	457	2640	ethanol	
β-apo-8’-carotinoic acid ethyl ester	cyclo-hexane	446	2540	ethanol	
6’-methyl-β-apo-6’-carotene-6’-one	*n*-hexane	468	2745	T/CH (1+4, v/v)	
*DL*-α-tocopherol	ethanol	292	75.8	ethanol	

T/CH, toluene+cyclohexane

The compounds were analyzed on FRAP and αTEAC activity in a V-530 spectrophotometer (JASCO, Groß-Umstadt, Germany) using half-micro cuvettes (1.5 mL, polystyrene; Plastibrand, Wertheim, Germany). A microplate reader FluoStar Optima (BMG Labtech, Offenburg, Germany) was used to analyze ROO^●^ radical scavenging activity in the CL assay. The antioxidant activity was calculated using a dose-response curve for α-tocopherol (approx. 5-250 µM) in *n*-hexane (for αTEAC and FRAP assay) or in TBME+DMSO (1+9, v/v) for CL assay, respectively [63]. The pure solvents were used as blank in the specific assay. The antioxidant activity of BC and its metabolites in each assay was calculated as mol α-tocopherol equivalents (α-TE)/mol compound.

### Determination of antioxidant activity

αTEAC, FRAP and CL assay to assess the antioxidant activity of BC isomers and its metabolites were done as described by our research group [34]. αTEAC assay was performed by mixing ABTS^●+^ working solution with solutions of BC isomers, its metabolites or with α-tocopherol standard. Thereafter, the mixture was completely transferred into cuvettes and centrifuged. Finally, the absorbance of the lower phase (ABTS layer) was measured at 734 nm. To assess the FRAP activity of these lipophilic compounds, solutions of α-tocopherol standard, BC isomer or metabolite were mixed with FRAP reagent. After transferring the mixed solution into cuvettes, and subsequent centrifugation, the absorbance of the aqueous layer was measured at 595 nm. To quantify the ROO^●^ radical scavenging activity of the BC compounds, a CL assay was performed, using luminol as CL dye and AAPH as ROO^●^ generator [69]. The assay was carried out in white 96-well Lumitrac micro plates (Greiner Bio-One, Frickenhausen, Germany). Luminol solution (in DMSO+borax buffer), solution of BC compound or α-tocopherol standard in TBME+DMSO (9+1, v/v), were combined in the wells of the micro plate. After addition of AAPH solution, the instrument was started to record the luminescence signals [34].

### Statistics

All analyses were performed in triplicate at four different concentrations of each BC compound (1-20 µmol/L). Differences of the antioxidant activity between (*all-E*)-β-carotene, its (*Z*)-isomers and its metabolites were calculated using one way analysis of variance (ANOVA) with Student-Newman-Keuls *post-hoc* procedure, with a level of significance at *p* < 0.05 (SPSS for Windows, version 18.0; SPSS Inc., Chicago, IL).

## References

[1] Maiani G., Caston M.J., Catasta G., Toti E., Cambrodon I.G., Bysted A., Granado-Lorencio F., Olmedilla-Alonso B., Knuthsen P., Valoti M., Böhm V., Mayer-Miebach E., Behsnilian D., Schlemmer U. (2009). Carotenoids: actual knowledge on food sources, intakes, stability and bioavailability and their protective role in human. Mol. Nutr. Food Res..

[2] Krinsky N.I., Johnson E.J. (2005). Carotenoid actions and their relation to health and disease. Mol. Aspects Med..

[3] Rodriguez-Amaya D.B., Kimura M., Godoy H.T., Amaya-Farfan J. (2008). Updated Brazilian database on food carotenoids: Factors affecting carotenoid composition. J. Food Compos. Anal..

[4] Zechmeister L., Tuzson P. (1938). Isomerization of carotenoids. Biochem. J..

[5] Castenmiller J.J., West C.E. (1998). Bioavailability and bioconversion of carotenoids. Annu. Rev. Nutr..

[6] Failla M.L., Thakkar S.K., Kim J.Y. (2009). *In vitro* bioaccessibility of β-carotene in orange fleshed sweet potato (*Ipomoea batatas, Lam.*). J. Agric. Food Chem..

[7] Deming D.M., Erdman J.W. (1999). Mammalian carotenoid absorption and metabolism. Pure Appl. Chem..

[8] Khachik F., Spangler C.J., Smith J.C., Canfield L.M. (1997). Identification, quantification, and relative concentrations of carotenoids and their metabolites in human milk and serum. Anal. Chem..

[9] Stahl W., Schwarz W., Sundquist A.R., Sies H. (1992). *cis-trans* isomers of lycopene and β-carotene in human serum and tissues. Arch. Biochem. Biophys..

[10] Voutilainen S., Nurmi T., Mursu J., Rissanen T.H. (2006). Carotenoids and cardiovascular health. Am. J. Clin. Nutr..

[11] Agte V., Tarwadi K. (2010). The importance of nutrition in the prevention of ocular disease with special reference to cataract. Ophthalmic Res..

[12] Kubo A., Corley D.A., Jensen C.D., Kaur R. (2010). Dietary factors and the risks of oesophageal adenocarcinoma and Barrett's oesophagus. Nutr. Res. Rev..

[13] Glauert H.P., Calfee-Mason K., Stemm D.N., Tharappel J.C., Spear B.T. (2010). Dietary antioxidants in the prevention of hepatocarcinogenesis: A review. Mol. Nutr. Food Res..

[14] Cranganu A., Camporeale J. (2009). Nutrition aspects of lung cancer. Nutr. Clin. Pract..

[15] Riccioni G. (2009). Carotenoids and cardiovascular disease. Curr. Atheroscler. Rep..

[16] Di Mascio P., Kaiser S., Sies H. (1989). Lycopene as the most efficient biological carotenoid singlet oxygen quencher. Arch. Biochem. Biophys..

[17] Levin G., Mokady S. (1994). Antioxidant activity of 9-*cis* compared to all-*trans* β-carotene *in vitro*. Free Radic. Biol. Med..

[18] Levin G., Yeshurun M., Mokady S. (1997). *In vivo* antiperoxidative effect of 9-*cis* β-carotene compared with that of the all-*trans* isomer. Nutr. Cancer.

[19] Böhm V., Puspitasari-Nienaber N.L., Ferruzzi M.G., Schwartz S.J. (2002). Trolox equivalent antioxidant capacity of different geometrical isomers of α-carotene, β-carotene, lycopene, and zeaxanth. J. Agric. Food Chem..

[20] Patrick L. (2000). Beta-carotene: the controversy continues. Altern. Med. Rev..

[21] Palozza P., Simone R., Mele M.C. (2008). Interplay of carotenoids with cigarette smoking: implications in lung cancer. Curr. Med. Chem..

[22] Leneberger M.G., Engeloch-Jarret C., Woggon W.-D. (2001). The reaction mechanism of the enzyme-catalyzed central cleavage of β-carotene in retinal. Angew. Chem., Int. Ed..

[23] Yeum K.J., Russell R.M. (2002). Carotenoid bioavailability and bioconversion. Annu. Rev. Nutr..

[24] von Lintig J., Hessel S., Isken A., Kiefer C., Lampert J.M., Voolstra O., Vogt K. (2005). Towards a better understanding of carotenoid metabolism in animals. Biochim. Biophys. Acta.

[25] Wang X.D., Tang G.W., Fox J.G., Krinsky N.I., Russell R.M. (1991). Enzymatic conversion of β-carotene into β-apo-carotenals and retinoids by human, monkey, ferret, and rat tissues. Arch. Biochem. Biophys..

[26] Nagao A. (2004). Oxidative conversion of carotenoids to retinoids and other products. J. Nutr..

[27] Ho C.C., de Moura F.F., Kim S.H., Clifford A.J. (2007). Excentral cleavage of β-carotene *in vivo* in a healthy man. Am. J. Clin. Nutr..

[28] Weedon B.C.L., O. Isler (1976). II. Occurence. Carotenoids.

[29] Barua A.B., Olson J.A. (2000). β-Carotene is converted primarily to retinoids in rats *in vivo*. J. Nutr..

[30] Alija A.J., Bresgen N., Sommerburg O., Langhans C.D., Siems W., Eckl P.M. (2005). Cyto- and genotoxic potential of β-carotene and cleavage products under oxidative stress. Biofactors.

[31] Mortensen A., Skibsted L.H., Truscott T.G. (2001). The interaction of dietary carotenoids with radical species. Arch. Biochem. Biophys..

[32] Lavy A., Ben Amotz A., Aviram M. (1993). Preferential inhibition of LDL oxidation by the all-*trans* isomer of β-carotene in comparison with 9-*cis* β-carotene. Eur. J. Clin. Chem. Clin. Biochem..

[33] Miller N.J., Sampson J., Candeias L.P., Bramley P.M., Rice-Evans C.A. (1996). Antioxidant activities of carotenes and xanthophylls. FEBS lett..

[34] Müller L., Fröhlich K., Böhm V. (2010). Comparative antioxidant activities of carotenoids measured by ferric reducing antioxidant power (FRAP), ABTS bleaching assay (αTEAC), DPPH assay and peroxyl radical scavenging assay. Food Chem..

[35] Böhm H., Boeing H., Hempel J., Raab B., Kroke A. (1998). Flavonols, flavones, and anthocyanins as native antioxidants of food and their possible role in the prevention of chronic diseases. Z. Ernährungswiss..

[36] Halliwell B., Rafter J., Jenner A. (2005). Health promotion by flavonoids, tocopherols, tocotrienols, and other phenols: direct or indirect effects? Antioxidant or not?. Am. J. Clin. Nutr..

[37] El-Agamey A., Lowe G.M., McGarvey D.J., Mortensen A., Phillip D.M., Truscott T.G., Young A.J. (2004). Carotenoid radical chemistry and antioxidant/pro-oxidant properties. Arch. Biochem. Biophys..

[38] Kamal-Eldin A., Appelqvist L.A. (1996). The chemistry and antioxidant properties of tocopherols and tocotrienols. Lipids.

[39] Young A.J., Lowe G.M. (2001). Antioxidant and prooxidant properties of carotenoids. Arch. Biochem. Biophys..

[40] Guo W.H., Tu C.Y., Hu C.H. (2008). *Cis-trans* isomerizations of β-carotene and lycopene: a theoretical study. J. Phys. Chem..

[41] Ceron-Carrasco J.P., Bastida A., Zuniga J., Requena A., Miguel B. (2009). Density functional theory study of the stability and vibrational spectra of the β-carotene isomers. J. Phys. Chem..

[42] Yoshida H., Kisugi R. (2010). Mechanisms of LDL oxidation. Clin. Chim. Acta.

[43] Pulido R., Bravo L., Saura-Calixto F. (2000). Antioxidant activity of dietary polyphenols as determined by a modified ferric reducing/antioxidant power assay. J. Agric. Food Chem..

[44] Benzie I.F., Strain J.J. (1996). The ferric reducing ability of plasma (FRAP) as a measure of "antioxidant power": the FRAP assay. Anal. Biochem..

[45] Spiteller G. (2006). Peroxyl radicals: inductors of neurodegenerative and other inflammatory diseases. Their origin and how they transform cholesterol, phospholipids, plasmalogens, polyunsaturated fatty acids, sugars, and proteins into deleterious products. Free Radic. Biol. Med..

[46] Foti M.C., Amorati R. (2009). Non-phenolic radical-trapping antioxidants. J. Pharm. Pharmacol..

[47] Woodall A.A., Lee S.W.-M., Weesie R.J., Jackson M.J., Britton G. (1997). Oxidation of carotenoids by free radicals: relationship between structure and reactivity. Biochim. Biophys. Acta.

[48] Burton G.W., Ingold K.U. (1984). β-Carotene: an unusual type of lipid antioxidant. Science.

[49] Terao J. (1989). Antioxidant activity of β-carotene-related carotenoids in solution. Lipids.

[50] Tsuchiya M., Scita G., Freisleben H.J., Kagan V.E., Packer L. (1992). Antioxidant radical-scavenging activity of carotenoids and retinoids compared to α-tocopherol. Meth. Enzym..

[51] Woodall A.A., Britton G., Jackson M.J. (1997). Carotenoids and protection of phospholipids in solution or in liposomes against oxidation by peroxyl radicals: Relationship between carotenoid structure and protective ability. Biochim. Biophys. Acta.

[52] Galano A. (2007). Relative antioxidant efficiency of a large series of carotenoids in terms of one electron transfer reactions. J. Phys. Chem. B.

[53] Naguib Y.M.A. (2000). Antioxidant activities of astaxanthin and related carotenoids. J. Agric. Food Chem..

[54] Huang D., Ou B., Prior R.L. (2005). The chemistry behind antioxidant capacity assays. J. Agric. Food Chem..

[55] Tabart J., Kevers C., Pincemail J., Defraigne J.-O., Dommes J. (2009). Comparative antioxidant capacities of phenolic compounds measured by various tests. Food Chem..

[56] Gradelet S., Leclerc J., Siess M.H., Astorg P.O. (1996). β-apo-8'-Carotenal, but not β-carotene, is a strong inducer of liver cytochromes P4501A1 and 1A2 in rat. Xenobiotica.

[57] Salgo M.G., Cueto R., Winston G.W., Pryor W.A. (1999). β-Carotene and its oxidation products have different effects on microsome mediated binding of benzo[a]pyrene to DNA. Free Radic. Biol. Med..

[58] Siems W., Sommerburg O., Schild L., Augustin W., Langhans C.D., Wiswedel I. (2002). β-Carotene cleavage products induce oxidative stress *in vitro* by impairing mitochondrial respiration. FASEB J..

[59] Siems W., Wiswedel I., Salerno C., Crifo C., Augustin W., Schild L., Langhans C.D., Sommerburg O. (2005). β-Carotene breakdown products may impair mitochondrial functions - potential side effects of high-dose β-carotene supplementation. J. Nutr. Biochem..

[60] Siems W., Salerno C., Crifo C., Sommerburg O., Wiswedel I. (2009). β-Carotene degradation products - formation, toxicity and prevention of toxicity. Forum Nutr..

[61] Marques S.A., Loureiro A.P., Gomes O.F., Garcia C.C., Di Mascio P., Medeiros M.H. (2004). Induction of 1,N(2)-etheno-2'-deoxyguanosine in DNA exposed to β-carotene oxidation products. FEBS lett..

[62] Carail M., Caris-Veyrat C. (2006). Carotenoid oxidation products: from villain to saviour?. Pure Appl. Chem..

[63] Müller L., Theile K., Böhm V. (2010). *In vitro* antioxidant activity of tocopherols and tocotrienols and comparison of vitamin E concentration and lipophilic antioxidant capacity in human plasma. Mol. Nutr. Food Res..

[64] Jimenez-Alvarez D., Giuffrida F., Vanrobaeys F., Golay P.A., Cotting C., Lardeau A., Keely B.J. (2008). High-throughput methods to assess lipophilic and hydrophilic antioxidant capacity of food extracts *in vitro*. J. Agric. Food Chem..

[65] Olmedilla B., Granado F., Rojas-Hidalgo E., Blanco I. (1990). A rapid separation of ten carotenoids, three retinoids, α-tocopherol and D-α-tocopherol acetate by high performance liquid chromatography and its application to serum and vegetable samples. J. Liq. Chromatogr..

[66] Schierle J., Härdi W., Faccin N., Bühler I., Schüep W., Britton G., Liaaen-Jensen S., Pfander H. (1995). Example 8: Geometrical isomers of β,β-carotene. A rapid routine method for quantitative determination. Carotenoids.

[67] Naumann C., Bassler R. (1976). Die chemische Untersuchung von Futtermitteln.

[68] Franke A.A., Murphy S.P., Lacey R., Custer L.J. (2007). Tocopherol and tocotrienol levels of foods consumed in Hawaii. J. Agric. Food Chem..

[69] Lissi E., Pascual C., Del Castillo M.D. (1992). Luminol luminescence induced by 2,2'-azo-bis(2-amidinopropane) thermolysis. Free Radic Res. Comm..

